# An evolutionary game analysis of digital transformation of multiagents in digital innovation ecosystems

**DOI:** 10.1371/journal.pone.0289011

**Published:** 2023-07-21

**Authors:** Baotong Liu, Hua Zou, Hao Qin, Huimin Ji, Yongquan Guo

**Affiliations:** College of Management, Shenyang University of Technology, Tiexi District, Shenyang, PR China; University of Cagliari: Universita degli Studi Di Cagliari, ITALY

## Abstract

In an innovation ecosystem, the digital transformation decisions and game mechanisms of entities are paramount issues to be studied. Consequently, this study constructs a digital transformation SD evolutionary game model based on expectancy theory and Lyapunov’s first law to address the above issues. The results demonstrate the following: (1) Digital technology empowerment benefits, spillover effects, and supervision benefits are positively correlated with the willingness of the three players to engage in digital transformation; (2) Regardless of how the initial will of the players changes, the decision of the evolutionary game system is ultimately stable in (empower, transform, supervise). Compared with governments, platform centers, and nodal enterprises have a stronger will for digital transformation. However, the governments’ will is the key to the convergence speed of the game system to the equilibrium point. (3) If the static/dynamic spillover effect can cover the transformation loss, even if the transformation profits of nodal enterprises are negative, nodal enterprises will still choose the game strategy of "transformation". When the government subsidies are less than the initial value of 2, the game system has two possible strategy choices: (empower, nontransform, nonsupervise) and (empower, transform, supervise). As such, this study can fill the research gaps and address the barriers to digital transformation among stakeholders.

## Introduction

The emergence of the digital age can fundamentally change the nature and structure of products and services and generate new paradigms and new ways of value creation among innovation agents. Therefore, everything in human society can be digitized [1], which has triggered scholars to consider digital innovation ecosystems (DIEs). Some acronyms are defined in Table 1. Scholar Chao Zhang [2] divided DIE into innovation-oriented and digital-empowered. Innovation-oriented DIE (i.e., "DIE Type I") refers to the ecosystem in which digital actors engage in digital innovation, commercialization, and application of achievements. Digital-empowered innovation ecosystems (i.e., "DIE Type II") refer to the innovation ecosystem formed in the process of digital transformation of innovation actors, structures, policies, and methods, which realizes deep integration of digitalization and the value cocreation process of innovation actors. Therefore, this paper chooses the latter as the conceptual and theoretical boundary of DIE. Building a digital innovation ecosystem helps to expand the business boundaries of organizations and achieve digital value co-creation. Companies such as Apple, Huawei, and Ali see this as a strategic goal to maintain their core competencies [3]. In the formation process, digital transformation (DT) not only impacts national and regional economies at the macro level [4] but also creates new market opportunities and innovation impetus for companies at the micro level [5]. The emergence of DIE is inextricably linked to DT. Despite the general agreement in academia and practice that research on DIE is highly important and promising, existing research is still at an early stage. These studies mainly focus on concepts [6–9], evolutionary patterns [10, 11], and governance mechanisms [12, 13] and are often conducted by qualitative methods such as literature reviews, case studies, and grounded theory.

**Table 1:** Acronyms.

*DIE*(*s*)	digital innovation ecosystem(s)
*DT*(*s*)	digital transformation(s)
*PC*(*s*)	platform center(s)
*NE*(*s*)	nodal enterprise(s)
*GA*(*s*)	government agency(s)

However, they ignore the premise that the DT of an innovation ecosystem is subject to the influence of the transformation willingness and interaction relationships of each actor within it. And, limited by the gap between dreams and reality, some managers report that 70% of digital transformation projects end up in failure [5]. In a typical case of failed transformation, General Electric (GE), hired the best software developers in Silicon Valley and invested more than $2 billion to become a software giant [14], but this caused excessive internal financial pressure and ultimately failed the transformation. In contrast, Siemens AG redefined its customer value chain, adjusted its original operating model and entrepreneurial culture in the emerging digital industrial market, and smoothly transitioned into the digital context [15]. For this reason, it is an important prerequisite for building a DIE to explore the evolutionary laws of digital transformation strategies and their decision influencing factors. Nambisan [16] believes that the decision-making of enterprises for DT is mainly subject to factors such as enterprise openness, profit orientation, digital technology creativity, core enterprise support, and government guidance. Therefore, we consider the above factors and analyze the evolutionary mechanisms of DT strategies of platform centers (PCs) and nodal enterprises (NEs) with the participation of government agencies (GAs) by combining evolutionary game models and knowledge of system dynamics. We then explore the drivers of the tripartite players to build DIE and conduct a simulation analysis. These are fundamental to the stable development of DIE. The main research questions motivating this study are as follows:

**RQ1:** How do the tripartite players choose DT strategies as they evolve over time?
**RQ2:** What are the key factors influencing tripartite players’ decision-making?
**RQ3:** How do changes in key factors affect the evolutionary outcome of the game system?

This paper makes the following contributions: (1) This paper studies the evolutionary mechanisms of DT of PC, NE, and GA in digital innovation ecosystems, which enriches the theoretical basis of DT and addresses the barriers to DT among players. (2) This paper explores the effect of government regulation on the digital transformation of players, which helps governments scientifically use policy and regulatory tools to confront the challenges of DT. (3) This paper introduces a system dynamics evolutionary game rate variable-based in-tree model to clarify the feedback mechanism of players’ decision-making behavior in DT under different scenarios.

## Literature review

### Digital innovation ecosystems

In the context of the digital economy, digitization promotes the development and popularization of digital innovations such as virtual reality technology, online services, and the sharing economy [17]. Thus, the innovation ecosystem has a digital character, which in turn gives rise to the DIE. Li showed that in the field of agriculture, the components of DIE include internal architect energy groups, external architect regulation groups, and digital agricultural innovation habitats, thus forming an evolutionary path of "environments—subjects—innovation chains—innovation networks" [11]. Yang et al. adopted the fsQCA method to study the influence of system participants and their relationships on the technical performance and financial performance of core enterprises [18]. Chu et al. used the conceptual model method to construct the operation mechanism of the digital intelligence empowerment innovation ecosystem, including the resource orchestration mechanism, knowledge value-added mechanism, openness mechanism, symbiotic evolution mechanism, technology-driven mechanism, flexible mechanism, performance feedback mechanism and support guarantee mechanism [19]. Shao et al. adopted a single-case study method to analyze in depth the formation process of digitally empowered innovation ecosystems, i.e., Digital technology empowers the action subjects in the system through resources, psychology, and structure, continuously forming information management and shared collaboration models, enabling enterprises to achieve leapfrog development from monolithic to diversified to systemic [20]. Shan et al. found that when the dominant players adopt the incentive-sharing competitive transformation strategy, each player can easily form a mutual competitive symbiosis, and the comprehensive benefits of data resources can be optimized in the digital innovation ecosystem [21]. Lin and Lu conducted an analysis based on the NCA and QCA approaches and identified digital enterprises, governments, universities and research institutions, digital innovation infrastructure, digital talent, and financial services as key components of the digital innovation ecosystem and found that the combination of these elements is conducive to improving regional innovation performance [22]. Gupt et al. proposed through text mining techniques that in the current matured e-commerce era, a well-known priority area for Digital Business Ecosystems applications is the use of (software) bots or agents to replace less efficient human interfaces [23]. From a systems transformation perspective, G et al. outlined a focal firm became the orchestrator of digital transformation amongst other interdependent actors in its business ecosystem [24].

### Digital transformation

Based on the literature, we can see that the articles on DT focus on four main aspects: conceptual construction, implementation path, problems, and countermeasures. Vial defined DT as the process of optimizing the development of the real economy by combining information, computing, communication, and connectivity technologies to change the fundamental attributes of entities [25]. Chulu and Ling demonstrated that platform centers dominate the digital transformation process of systems because they are developers of digital technologies and owners of data resources, and they provide support to subjects (i.e., ’nodal enterprises’) that use digital technologies and resources for application-side innovation or digital transformation [9]. Teece illustrated that digital technologies dominate the transformation and upgrading of enterprises, resulting in static spillover effects and dynamic spillover effects. Static spillover effects are the positive externalities of technology standards that provide the ground rules for digital technology empowerment; dynamic spillover effects are the technological innovation opportunities for other agents brought about by the continuous iterative upgrading in technology application [26]. Zhou sorted out the realization path of DT of enterprises based on micro survey data. Specifically, enterprises should objectively examine their own characteristics and resource endowments, invest in talent, technologies, data, and other elements, and subsequently choose to purchase services, cooperate with external parties, or carry out the transformation independently [27]. Qi and Xiao found that user value-led and alternative competition fundamentally motivates the innovation of business management models in the context of the digital economy and suggested that enterprises should establish awareness of DT and develop early implementation strategies [28]. Chen and Wang studied the case of Rococo Group and the Nailing platform to cooperate in upgrading and built a "dependent upgrading" model for ecological participants to promote DT with the help of platform enterprises while sorting out three development stages: mutual integration, symbiosis and self-development [29]. Matthew et al. discussed the barriers that enterprises face in the process of DT, such as the lack of technology, higher investment, insufficient data resources, and the difficulty of integrating the value chain, through a literature review approach [30]. Carmelo et al. argued that when companies have too many things to digitally transform, there is a lack of prioritization and ambiguity in organizational authority and responsibility [31]. Dong et al. demonstrated that companies need to focus on the importance of investing in digital green innovation projects to improve their green competitiveness [32]. Chi et al. measured that DT is a key necessity to improve firms’ innovation performance based on NCA and SEM methods and suggested that firms should increase IT capabilities and capital investment and that governments should actively optimize the institutional environment for DT, increase investment in resources such as 5G, AI and data centers and guide firms to learn from successful transformation experiences [33]. Rocha et al. case study suggested that organizational ecosystems will become increasingly open and collaborative as enterprises undergo digital transformation. and actively recognizes the important role of big data in enterprise analytics, forecasting, and decision-making [34].

### Evolutionary game model

As evolutionary game theory has been studied in depth, scholars have applied it to the field of management. Since the model has the following advantages: it can demonstrate the dynamic evolution of a group over time, select the optimal strategy and solve the problem that actors are not fully rational [35], it has been widely used in the study of corporate strategy choice, supply chain management, and the evolution of system structures [36]. Tan and Zhao applied the model to study the issue of innovation protection strategies and the choice of follow-up strategies for innovation ecosystem subjects in a differentiated environment and analyzed the influence of institutional, ecological and technological factors on the evolutionary equilibrium stability, and evolutionary stabilization strategies of innovation ecosystems [37]. He et al. analyzed the strategy choices of cloud manufacturing service integrators and suppliers in the knowledge-sharing incentive process, derived evolutionary equilibrium strategies under different parameter constraints, and conducted an evolutionary stability analysis of the dynamic cooperation process of the knowledge-sharing incentive [38]. Wang et al. constructed a tripartite evolutionary model to examine the effects of mandatory and market-driven environmental regulations on the diffusion of green technology innovations in manufacturing firms [39]. Jin constructed a heterogeneous two-entity game model to identify the influencing factors, stability strategies, and inner laws of digital transformation in accounting firms [40]. Huang et al. applied the model to explore the development process of digital transformation in the cultural industry [41]. Zhang et al. constructed an evolutionary game model to improve the transaction efficiency and information transmission stability of the blockchain and measured the success rate and cost under different strategies, and the experimental results showed that the proposed model can be used to improve a channel’s stability and keep it in a good cooperative stable state [42, 43]. Meng et al. used an evolutionary game to measure the difference in strategies between mandatory and voluntary vaccination based on the reality of COVID-19 to provide better vaccination strategies for government decisions to control the spread of infectious diseases [44]. As seen, evolutionary game models can show and solve realistic problems very well. However, most of the existing studies conduct bipartite games, lack the application of tripartite evolutionary game models and the combination of other models, and few studies have explored the dynamic evolutionary process of digital transformation in the context of digital innovation ecosystems.

### Summary

In summary, on the one hand, studies on DIE focus on conceptualization, structure, and operating mechanisms, which provide a more complete theoretical foundation and framework for subsequent studies; on the other hand, studies on digital transformation focus on transformation paths, obstacles, and countermeasures, which provide theoretical references for this paper. At the same time, evolutionary game models are often used singularly and lack a combination with other models, while existing studies mostly focus on the strategic choices of two subjects and lack coevolutionary analysis of three or more subjects. Therefore, to fill the abovementioned gaps, the innovations of this paper are as follows: (1) This paper constructs a digital transformation tripartite players SD evolutionary game rate variable based on an in-tree model and considers the dynamic changes in parameters such as digital technology empowerment revenue and cost, static/dynamic spillover effect based on system dynamics and evolutionary game theory. (2) We use the digital innovation ecosystem as the research context and conduct simulations combined with the actual situation of China’s digital transformation to make the results more generalizable. (3) This study integrates the effects of various parameters on the strategic evolutionary equilibrium of the digital transformation system and explores the dynamic evolutionary process of the decision-making behavior of the three players and their feedback mechanisms. It also clarifies the roles and values of the three players in DT.

## Model construction

### Game logic

**Fig 1:**
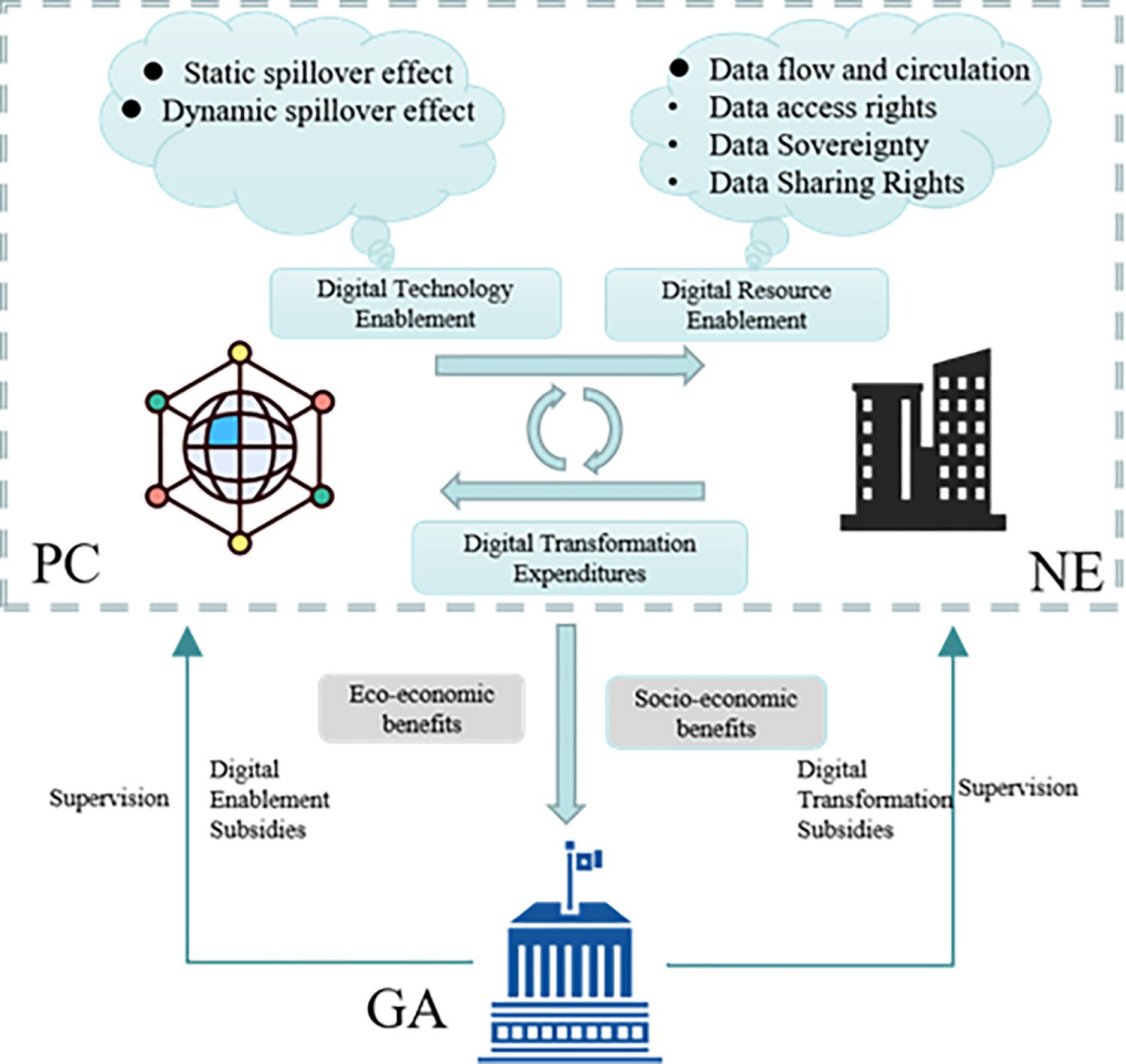
Logical relationship of the evolutionary game model of digital transformation.

In DIE, when NE is digitally transformed, they receive digital technology support, access to digital resources, transformation subsidies, and transformation revenues. PC provides technical support and data resources to NE through both digital technology empowerment and resource empowerment and subsequently generates static and dynamic spillover effects. GA formulates relevant laws, regulations and policies for supervision, and provides subsidies to PC and NE for digital transformation. Finally, GA can reap environmental and social benefits in the process of enterprise digital transformation. This paper constructs an evolutionary game model of PA and NE in the DIE with the participation of GA. The logical relationships among the three-game players are shown in Fig 1.

### Hypothesis

This paper is based on the following hypotheses:

**Hypothesis 1:** Participants. There are three participants, PC, NE, and GA, in the model, and they are all bounded rationality participants. PC and NE pursue maximum economic benefits from digital transformation. The government pursues maximum environmental and socioeconomic benefits.
**Hypothesis 2:** Strategy. In the DIE, PC has two behavioral options based on the measurement of empowerment gains and losses, with a strategy set of {empower, nonempower}; NE has two behavioral options after comparing the benefits of digital transformation with the benefits of traditional operations, with a strategy set of {transform, nontransform}; the flow and circulation of digital resources are restricted by laws and regulations, and GA plays a crucial role in this process. There are two behavioral options to promote the digital transformation of enterprises, with a strategy set of {supervise, nonsupervise}.
**Hypothesis 3:** Proportion. We let *x*(0≤*x*≤1) denote the proportion of the “empower” strategy, and (1-*x*) denote the proportion of the “nonempower” strategy. In addition, let *y*(0≤*y*≤1) and (1-*y*) denote the proportion of NE with a choice of “transform” and “nontransform”, respectively. Let *z*(0≤*z*≤1) and (1-*z*) denote the proportion of GA with a choice of “supervise” and “nonsupervise”, respectively. The parameters in the model are shown in Table 2.

**Table 2:** Description of major parameters.

Parameters	Descriptions
*R*0	When PC does not empower NE, the basic economic benefits are *R*0.
*R*1	*R*1 is the revenue earned by the PC for digital technology empowerment.
*C*1	*C*1 is the cost of digital technology empowerment by the PC.
*ω*1	*ω*1 is the coefficient of the static spillover effect generated by the digital technology empowerment of the PC,0≤*ω*1≤1.
*ω*2	*ω*2 is the coefficient of the dynamic spillover effect generated by the digital technology empowerment of the PC,0≤*ω*2≤1.
*R*2	*R*2 is the revenue generated by the digital resource empowerment of the PC.
*C*2	*C*2 is the cost of the PC’s digital resource empowerment by the PC.
*C*r	The risk cost is *C*r when the data sovereignty of the PC is violated.
*R*3	*R*3 is the economic benefit of the NE in the traditional way.
*R*4	*R*4 is the revenue earned by the NE for digital transformation.
*C*3	*C*3 is the cost of digital transformation for the NE.
Δ*Rg*	Δ*Rg* is the additional economic benefit to the GA from the transformation of the NE.
*Rg*	*Rg* is the environmental and socioeconomic benefits gained from GA supervision.
*Cg*	*Cg* is the cost of GA supervision.
*S*1	*S*1 are government subsidies for the digital empowerment of PC.
*S*2	*S*2 are government subsidies for the transformation of NE.

### Formulas

**Fig 2:**
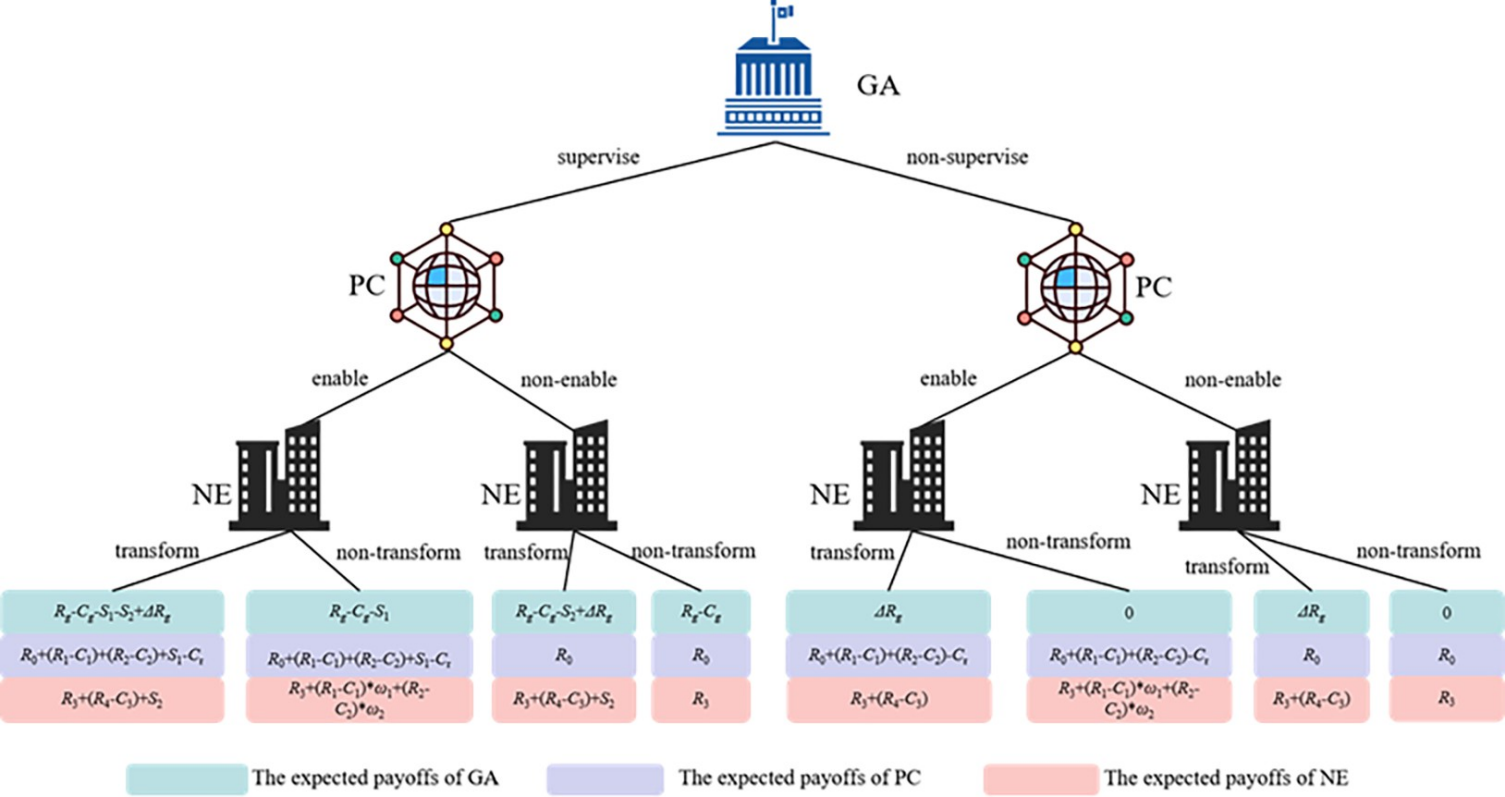
Game tree of government agencies (GAs), platform centers (PCs) and node enterprises (NEs).


Based on the above analysis and assumptions, the expected payoffs of the three players under different strategy choices are shown in the game tree in Fig 2. Thus, in the DIE, if the PC chooses "empower" or "nonempower", the expected payoffs are *E*_11_ and *E*_12_, respectively, and the average expected payoff is ${\bar{E}}_{1}$.

$$\begin{array}{l} E_{11}=y*z*(R_{0}-C_{2}-C_{r}-C_{1}+R_{1}+R_{2}+S_{1})-(y-1)*(z-1)*(C_{1}+C_{2}+C_{r}-R_{0}-R_{1}-R_{2})\\ -z*(y-1)*(R_{0}-C_{2}-C_{r}-C_{1}+R_{1}+R_{2}+S_{1})+y*(z-1)*(C_{1}+C_{2}+C_{r}-R_{0}-R_{1}-R_{2}) \end{array}$$ (1)

$$E_{12}=R_{0}*(y-1)*(z-1)-R_{0}*z*(y-1)-R_{0}*y*(z-1)+R_{0}*y*z$$ (2)

$${\bar{E}}_{1}=R_{0}-C_{1}*x-C_{2}*x-C_{r}*x+R_{1}*x+R_{2}*x+S_{1}*x*z$$ (3)

If the NE chooses "transform" or "nontransform", the expected payoffs are *E*_21_ and *E*_22_, respectively, and the average expected payoff is ${\bar{E}}_{2}$.


$$\begin{array}{l} E_{21}=x*z*(R_{3}-C_{3}+R_{4}+S_{2})-x*(z-1)*(R_{3}-C_{3}+R_{4})\\ -z*(x-1)*(R_{3}-C_{3}+R_{4}+S_{2})+(x-1)*(z-1)*(R_{3}-C_{3}+R_{4}) \end{array}$$ (4)

$$\begin{array}{l} E_{22}=x*(z-1)*[\omega_{1}*(C_{1}-R_{1})-R_{3}+\omega_{2}*(C_{2}-R_{2})]-R_{3}*z*(x-1)\\ +R_{3}*(x-1)*(z-1)-x*z*[\omega_{1}*(C_{1}-R_{1})-R_{3}+\omega_{2}*(C_{2}-R_{2})] \end{array}$$ (5)

$$\begin{array}{l} {\bar{E}}_{2}=R_{3}-C_{3}*y+R_{4}*y-C_{1}*\omega_{1}*x-C_{2}*\omega_{2}*x+R_{1}*\omega_{1}*x+R_{2}*\omega_{2}*x\\ +S_{2}*y*z+C_{1}*\omega_{1}*x*y+C_{2}*\omega_{2}*x*y-R_{1}*\omega_{1}*x*y-R_{2}*\omega_{2}*x*y \end{array}$$ (6)

If the GA chooses "supervise" or "nonsupervise", the expected payoffs are *E*_31_ and *E*_32_, respectively, and the average expected payoff is ${\bar{E}}_{3}$.


$$\begin{array}{l} E_{31}=y*(x-1)*(C_{g}-\Delta R_{g}-R_{g}+S_{2})+x*(y-1)*(C_{g}-R_{g}+S_{1})\\ -(C_{g}-R_{g})*(x-1)*(y-1)-x*y*(C_{g}-\Delta R_{g}-R_{g}+S_{1}+S_{2}) \end{array}$$ (7)

$$E_{32}=\Delta R_{g}*x*y‐\Delta R_{g}*(x‐1)*y$$ (8)

$${\bar{E}}_{3}=\Delta R_{g}*y-C_{g}*z+R_{g}*z-S_{1}*x*z-S_{2}*y*z$$ (9)

## Stability analysis

### PC stability analysis

#### Analysis based on the replicator dynamic equation

According to Eqs (1)–(3), the replicator dynamic equation of the PC is given as follows:

$$F(x)=\frac{dx}{dt}=x*\left(x-1\right)*\left(C_{1}+C_{2}+C_{r}-R_{1}-R_{2}-S_{1}*z\right)$$ (10)

The derivative of F(x) gives:

$$F'(x)=\frac{dF(x)}{dx}=(2x-1)(C_{1}+C_{2}+C_{r}-R_{1}-R_{2}-S_{1}*z)$$ (11)

We analyzed the evolutionary stability of the PC game strategies. When *F*(*x*) = 0, there are two cases: ① When $C_{1}+C_{2}+C_{r}-R_{1}-R_{2}-S_{1}*z=0$, take $z_{0}=(C_{1}+C_{2}+C_{R}-R_{1}-R_{2})/S_{1}$, i.e., *F*(*x*) = 0 when *z* = *z*_0_, which indicates that the game strategies of the PC are stable at this time, regardless of the value of *x*;

② When *z*≠*z*_0_ and *F*(*x*) = 0, *x* = 0 and *x* = 1 are the two stable points of the PC game strategy in the system.

**Fig 3:**
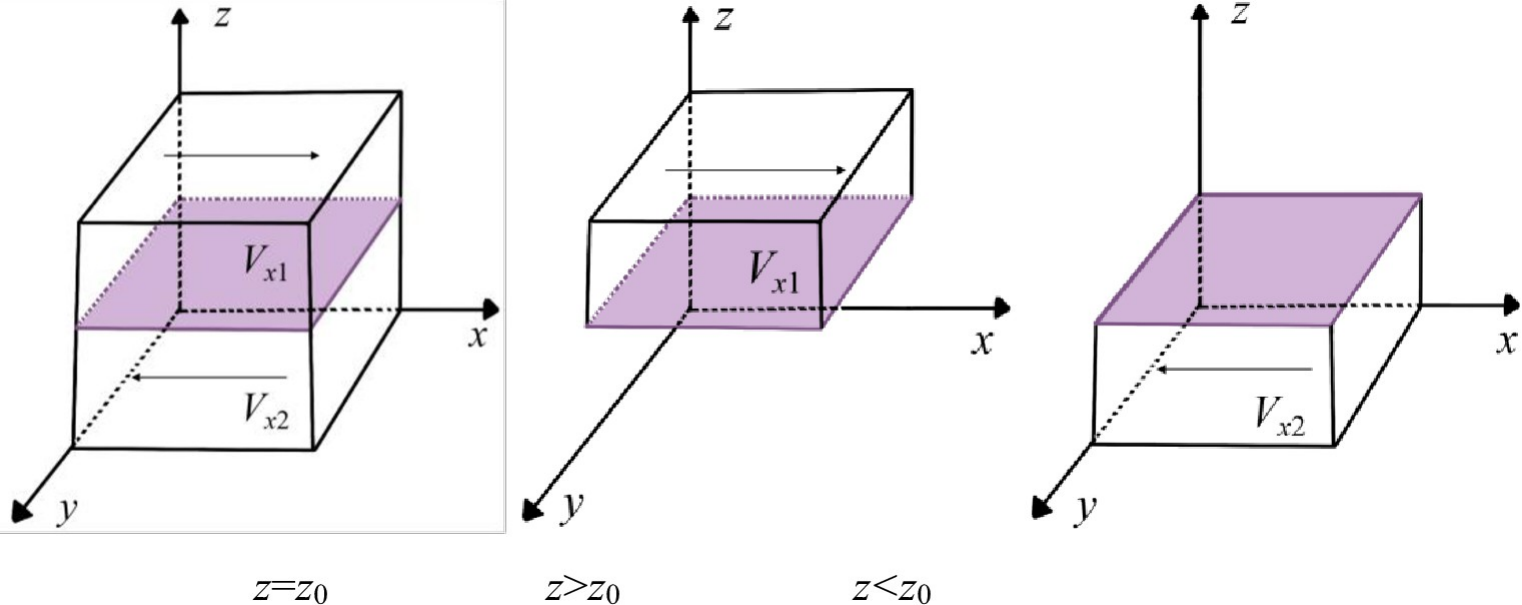
Dynamic evolution of PC decision-making.

According to the stability theory of differential equations, the PC must satisfy *F*(*x*) = 0, *dF*(*x*)/*dx*<0 if the "empower" strategy is in a stable state. When $G(z)=C_{1}+C_{2}+C_{r}-R_{1}-R_{2}-S_{1}*z$, $\partial G(z)/\partial z=-S_{1}<0$, so *G*(*z*) is a decreasing function about *z*. **Case 1:** When *z*>*z*_0_, *G*(*z*)<0, *F*′(*x*)|_*x* = 0_>0, *F*′(*x*)|_*x* = 1_<0, *x* = 1 is the evolutionary stabilization strategy (ESS) of the PC, and it tends to choose the "empower" game strategy. **Case 2:** When *z*<*z*_0_, *G*(*z*)>0, *F*′(*x*)|_*x* = 0_<0, *F*′(*x*)|_*x* = 1_>0, at this time, *x*= 0 is the ESS of the PC, and it tends to choose the "nonempower" game strategy. Based on the above analysis, the strategy evolution process of the PC is shown in Fig 3.

**Proposition 1:** When the PC’s decisions are initially located in the space *V*_*x*1_, *x* = 1 is the stable equilibrium point in *V*_*x*1_, namely, the PC’s game strategy gradually evolves in the direction of "empower". Therefore, when the PC’s digital empowerment revenues and government subsidies are greater than the costs, the PC’s game strategy will eventually stabilize to "empower" the digital transformation of NE over time.
**Proposition 2:** When the PC’s decisions are initially located in the space *V*_*x*2_, *x* = 0 is the stable equilibrium point in *V*_*x*2_, namely, the PC’s game strategy gradually evolves in the direction of "nonempower". Thus, when the PC’s digital empowerment cost is greater than the sum of revenue and government subsidies, over time, the PC’s game strategy will eventually stabilize to "nonempower" the digital transformation of NE.

#### Parameter analysis

As shown in Fig 3, when *C*_1_, *C*_2_, and *C*_*r*_ increase and the other parameters remain the same, *z*_0_ increases, and the cross-section moves upward. Therefore, *V*_*x*1_ decreases, and *V*_*x*2_ becomes larger. This indicates that PC has excessive digital empowerment costs, and the proportion of strategy choices that tend to be "nonempower" becomes larger. However, when *C*_1_, *C*_2_, and *C*_*r*_ decrease and other parameters remain the same, *z*_0_ decreases, and the cross-section moves downward. Therefore, *V*_*x*1_ becomes larger, and *V*_*x*2_ decreases. This shows that the cost of digital empowerment of the PC becomes progressively smaller, and the proportion of strategy choices that tend to be "empower" becomes larger.

### NE stability analysis

#### Analysis based on the replicator dynamic equation

According to Eqs (4)–(6), the replicator dynamic equation of the NE is given as follows:

$$F(y)=y*(1‐y)*(R_{4}-C_{3}+S_{2}*z+C_{1}*\omega_{1}*x+C_{2}*\omega_{2}*x-R_{1}*\omega_{1}*x-R_{2}*\omega_{2}*x)$$ (12)

The derivative of *F*(*y*) gives:

$$F^{\prime }(y)=\frac{dF(y)}{dy}=(1‐2y)(R_{4}-C_{3}+S_{2}*z+C_{1}*\omega_{1}*x+C_{2}*\omega_{2}*x-R_{1}*\omega_{1}*x-R_{2}*\omega_{2}*x)$$ (13)

We analyzed the evolutionary stability of the NE game strategies. When *F*(*y*) = 0, there are two cases:

① When $R_{4}-C_{3}+S_{2}*z+(C_{1}*\omega_{1}+C_{2}*\omega_{2}-R_{1}*\omega_{1}-R_{2}*\omega_{2})*x=0$, take $z_{0}=[C_{3}-R_{4}-(C_{1}*\omega_{1}+C_{2}*\omega_{2}-R_{1}*\omega_{1}-R_{2}*\omega_{2})*x]/S_{2}$, i.e., *F*(*y*) = 0 when *z* = *z*_0_, which indicates that the game strategies of the NE are stable at this time, regardless of the value of *y*;

② When *z*≠*z*_0_ and *F*(*y*) = 0, *y* = 0 and *y* = 1 are the two stable points of the NE game strategy in the system.

**Fig 4:**
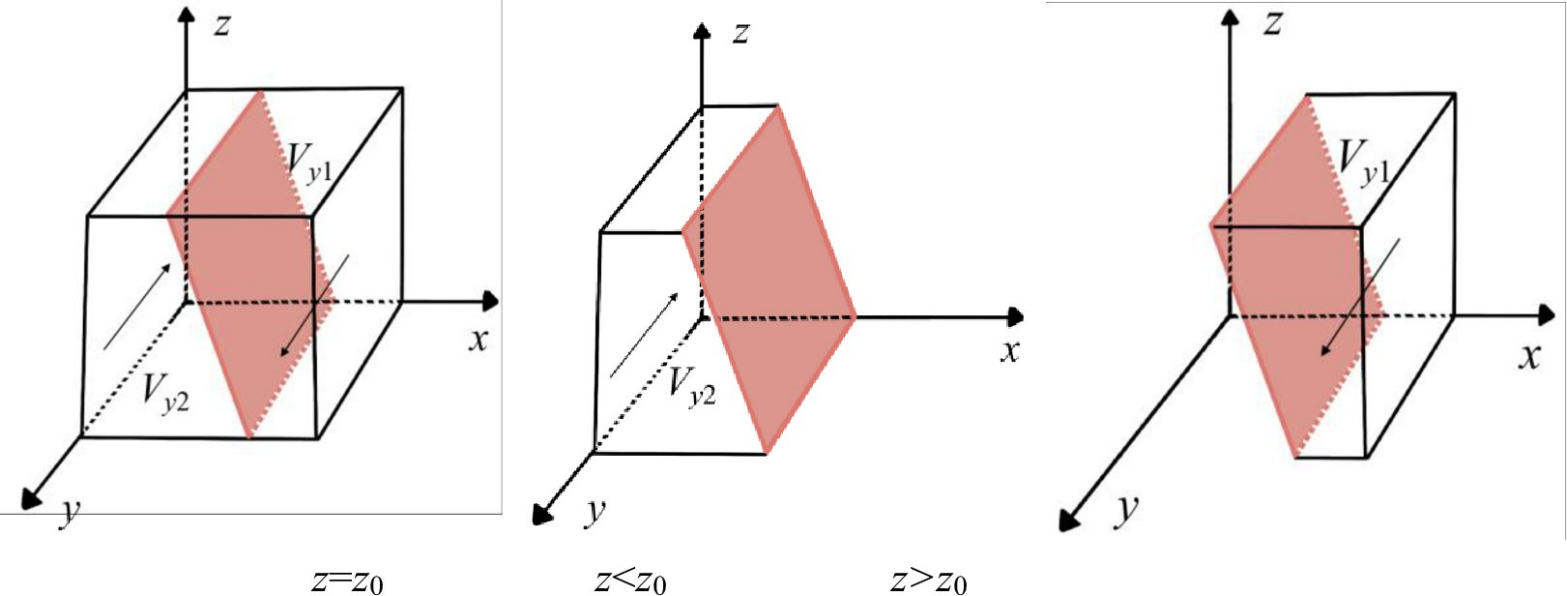
Dynamic evolution of NE decision-making.

According to the stability theory of differential equations, the NE must satisfy *F*(*y*) = 0, *dF*(*y*)/*dy*<0 if the "transform" strategy is in a stable state. Let $H(z)=R_{4}-C_{3}+S_{2}*z+(C_{1}*\omega_{1}+C_{2}*\omega_{2}-R_{1}*\omega_{1}-R_{2}*\omega_{2})*x$, then $\partial H(z)/\partial z=S_{2}>0$, and *H*(*z*) is an increasing function about *z*. **Case 1:** When *z*>*z*_0_, *H*(*z*)>0, *F*′(*y*)|_*y* = 0_>0, *F*′(*y*)|_*y* = 1_<0, at this time, *y* = 1 is the ESS of the NE, and it tends to choose the "transform" game strategy. **Case 2:** When *z*<*z*_0_, *H*(*z*)<0, *F*′(*y*)|_*y* = 0_<0, *F*′(*y*)|_*y* = 1_>0, at this time, *y* = 0 is the ESS of the NE, and it tends to choose the "nontransform" game strategy. Based on the above analysis, the strategy evolution process of the NE is shown in Fig 4.

**Proposition 3:** When the NE’s decisions are initially located in *V*_*y*1_, *y* = 1 is the stable equilibrium point in *V*_*y*1_, namely, the NE’s game strategy gradually evolves in the direction of "transform". Thus, when the spillover effect of the PC becomes larger or the profits of NE’s digital transformation become larger, the NE’s game strategy will eventually stabilize to "transform" over time.
**Proposition 4:** When the NE’s decisions are initially located in *V*_*y*2_, *y* = 0 is the stable equilibrium point in *V*_*y2*_, namely, the NE’s game strategy gradually evolves in the direction of "nontransform". Thus, when the spillover effect of the PC decreases or the profits of the NE digital transformation decrease, the NE’s game strategy will eventually stabilize to "nontransform" over time.

#### Parameter analysis

From Fig 4, when *C*_3_ decreases and the other parameters remain the same, *z*_0_ decreases, and the cross-section shifts to the left. Thus, *V*_*y2*_ decreases and *V*_*y*1_ becomes larger, indicating that the NE has excessive digital transformation costs, and the proportion of strategy choices tending to "transform" becomes larger. Conversely, NE’s strategy choices tend to be more "nontransform".

### GA stability analysis

#### Analysis based on the replicator dynamic equation

According to Eqs (7)–(9), the replicator dynamic equation of the NE is given as follows:

$$F(z)=z*(z-1)*(C_{g}-R_{g}+S_{1}*x+S_{2}*y)$$ (14)

The derivative of *F*(*z*) gives:

$$F^{\prime }(z)=\frac{dF(z)}{dz}=(2z-1)(C_{g}-R_{g}+S_{1}*x+S_{2}*y)$$ (15)

We analyzed the evolutionary stability of the GA game strategies. When *F*(*z*) = 0, there are two cases:

① When $C_{g}-R_{g}+S_{1}*x+S_{2}*y=0$, take $y_{0}=(C_{g}-R_{g}+S_{1}*x)/(-S_{2})$, i.e., *F*(*z*) = 0 when *y* = *y*_0_, which indicates that the game strategies of the GA are stable at this time, regardless of the value of *z*;

② When *y*≠*y*_0_ and *F*(*z*) = 0, *z* = 0 and *z* = 1 are the two stable points of the GA game strategy in the system.

**Fig 5:**
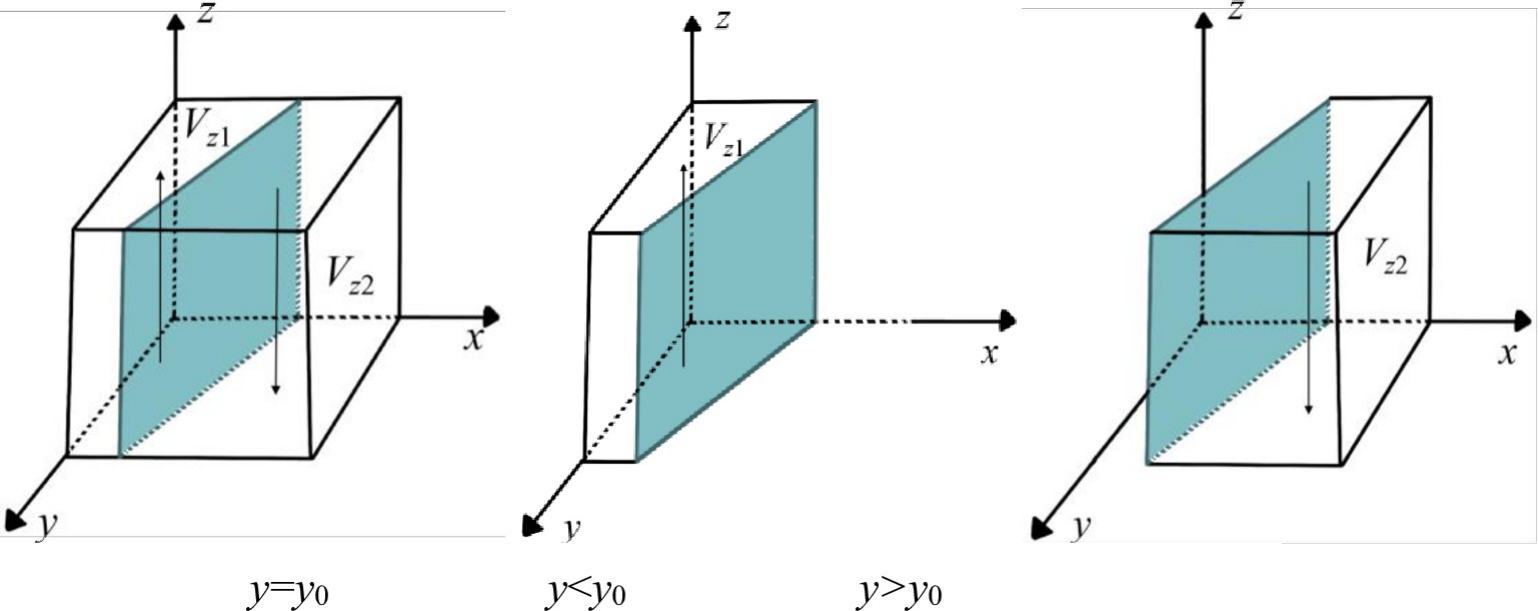
Dynamic evolution of GA decision-making.

According to the stability theory of differential equations, the GA must satisfy *F*(*z*) = 0, *dF*(*z*)/*dz*<0 if the "supervise" strategy is in a stable state. Let $G(y)=C_{g}-R_{g}+S_{1}*x+S_{2}*y$, then $\partial G(y)/\partial y=S_{2}>0$, and *G*(*y*) is an increasing function about *y*. **Case 1:** When *y*>*y*_0_, *G*(*y*)>0, *F*′(*z*)|_*z* = 0_<0, *F*′(*z*)|_*z* = 1_>0, at this time, *z* = 0 is the ESS of the GA, and it tends to choose the "nonsupervise" game strategy. **Case 2:** When *y*<*y*_0_, *G*(*y*)<0, *F*′(*z*)|_*z* = 0_>0, *F*′(*z*)|_*z* = 1_<0, at this time, *z* = 1 is the ESS of the GA, and it tends to choose the "supervise" game strategy. Based on the above analysis, the strategy evolution process of the GA is shown in Fig 5.

**Proposition 5:** When the GA’s decisions are initially located in *V*_*z*1_, *z* = 1 is the stable equilibrium point in *V*_*z*1_, namely, the GA’s game strategy gradually evolves in the direction of "supervise". Thus, when the supervision costs become lower or the social and environmental economic benefits become higher, the GA’s game strategy will eventually stabilize in the "supervise" over time.
**Proposition 6:** When the GA’s decisions are initially located in *V*_*z*2_, *z* = 0 is the stable equilibrium point in *V*_*z*2_, namely, the GA’s game strategy gradually evolves in the direction of "nonsupervise". Thus, when the supervision costs are higher or the social and environmental economic benefits become lower, the GA’s game strategy will eventually stabilize to "nonsupervise" over time.

#### Parameter analysis

From Fig 5, when *C*_*g*_ decreases and other parameters remain the same, *y*_0_ becomes larger, and the cross-section shifts to the right. Thus, *V*_*z2*_ decreases and *V*_*z*1_ becomes larger, which indicates that the GA has smaller supervision costs, and the proportion of strategy choices tending to "supervise" becomes larger. Conversely, GA’s strategy choices tend to be more "non supervise".

### Stability analysis of game system combination strategies

To find the balance of interests among PC, NE, and GA in the DIE, let *F*(*x*) = 0, *F*(*y*) = 0 and *F*(*z*) = 0 to obtain the Jacobian matrix of PC, NE, and GA as:

$$J=[\begin{array}{ccc} \left(2x-1)*(C_{1}+C_{2}+C_{r}-R_{1}-R_{2}-S_{1}*z\right) & 0 & -S_{1}*x*(x-1)\\ \left(1-y^{2})*(C_{1}*\omega_{1}+C_{2}*\omega_{2}-R_{1}*\omega_{1}-R_{2}*\omega_{2}\right) & \left(1-2y)*(R_{4}-C_{3}+S_{2}*z+C_{1}*\omega_{1}*x+C_{2}*\omega_{2}*x-R_{1}*\omega_{1}*x-R_{2}*\omega_{2}*x\right) & -S_{2}*y*(y-1)\\ S_{1}*z*(z-1) & S_{2}*z*(z-1) & \left(2z-1)*(C_{g}-R_{g}+S_{1}*x+S_{2}*y\right) \end{array}]$$

According to the Lyapunov indirect method, when the eigenvalues of the equilibrium points are all negative, the equilibrium point is an evolutionary stability strategy; otherwise, it is an unstable point. Hence, this paper has eight pure strategy equilibrium points in the game system, and their stability analysis is shown in Table 3. The eight equilibrium points are *E*_1_(0,0,0), *E*_2_(1,0,0), *E*_3_(0,1,0), *E*_4_(0,0,1), *E*_5_(1,1,0), *E*_6_(1,0,1), *E*_7_(0,1,1), and *E*_8_(1,1,1), and there exists one possible stable equilibrium point, namely, E8(1,1,1). It is shown that the game system is in stable equilibrium when the condition ①: $\left|R_{4}-C_{3}>|C_{1}*\omega_{1}+C_{2}*\omega_{2}-R_{1}*\omega_{1}-R_{2}*\omega_{2}\right|$ is satisfied, and the stable point is (1,1,1), which means that the game combination strategy is (empower, transform, supervise). Specifically, the PC chooses the digital empowerment strategy when the NE performs digital transformation, and the GA supervises the process. At this time, the game strategy of the system combination is optimal. For the PC, if the sum of their empowerment revenues and subsidies are higher than their empowerment costs and risk costs, their game strategies will stabilize to "empower" over time. For the NE, when the digital transformation revenues and subsidies are greater than the transformation costs, their game strategies will stabilize to "transform" over time. For the GA, when the environmental and socioeconomic benefits of supervision are larger than the costs of supervision and the number of subsidies, their game strategies will stabilize to "supervise" over time.

**Table 3:** Stability analysis of equilibrium points.

Equilibrium points	Eigenvalues			Symbols	State	Conditions
	*λ*_1_	*λ*_2_	*λ*_3_			
*E*_1_(0,0,0)	*R*_1_-*C*_2_-*C*_*r*_-*C*_1_+*R*_2_	*R*_4_-*C*_3_	*R*_*g*_-*C*_*g*_	(+,+,+)	Instability point	\
*E*_2_(1,0,0)	*C*_1_+*C*_2_+*C*_*r*_-*R*_1_-*R*_2_	*R*_4_-*C*_3_+*C*_1_*ω_1_+*C*_2_*ω_2_-*R*_1_*ω_1_-*R*_2_*ω_2_	*R*_*g*_-*C*_*g*_-*S*_1_	(-,+,+)	Instability point	①
*E*_3_(0,1,0)	*R*_1_-*C*_2_-*C*_*r*_-*C*_1_+*R*_2_	*C*_3_-*R*_4_	*R*_*g*_-*C*_*g*_-*S*_2_	(+,-,+)	Instability point	\
*E*_4_(0,0,1)	*R*_1_-*C*_2_-*C*_*r*_-*C*_1_+*R*_2_+*S*_1_	*R*_4_-*C*_3_+*S*_2_	*C*_*g*_-*R*_*g*_	(+,+,-)	Instability point	\
*E*_5_(1,1,0)	*C*_1_+*C*_2_+*C*_*r*_-*R*_1_-*R*_2_	*C*_3_-*R*_4_-*C*_1_*ω_1_-*C*_2_*ω_2_+*R*_1_*ω_1_+*R*_2_*ω_2_	*R*_*g*_-*C*_*g*_*-S*_1_-*S*_2_	(-,-,+)	Instability point	①
*E*_6_(1,0,1)	*C*_1_+*C*_2_+*C*_*r*_-*R*_1_-*R*_2_-*S*_1_	*R*_4_-*C*_3_+*S*_2_+*C*_1_*ω_1_+*C*_2_*ω_2_-*R*_1_*ω_1_-*R*_2_*ω_2_	*C*_*g*_-*R*_*g*_+*S*_1_	(-,+,-)	Instability point	①
*E*_7_(0,1,1)	*R*_1_-*C*_2_-*C*_*r*_-*C*_1_+*R*_2_+*S*_1_	*C*_3_-*R*_4_-*S*_2_	*C*_*g*_-*R*_*g*_+*S*_2_	(+,-,-)	Instability point	\
*E*_8_(1,1,1)	*C*_1_+*C*_2_+*C*_*r*_-*R*_1_-*R*_2_-*S*_1_	*C*_3_-*R*_4_-*S*_2_-*C*_1_*ω_1_-*C*_2_*ω_2_+*R*_1_*ω_1_+*R*_2_*ω_2_	*C*_*g*_-*R*_*g*_+*S*_1_+*S*_2_	(-,-,-)	*ESS*②	①

①:*R*_4_-*C*_3_>|*C*_1_*ω_1_+*C*_2_*ω_2_-*R*_1_*ω_1_-*R*_2_*ω_2_|.

②:ESS is the evolutionary stable strategy of the system.

## Simulation analysis

By constructing a tripartite evolutionary game model, we discussed the stability of a single player and system combination game strategy under different conditions. However, it does not clearly show the specific process of system evolution and the internal influence mechanism. Accordingly, to verify the rationality of the propositions of the evolutionary game model and the sensitivity of the parameters, based on the above analysis, we combined the knowledge of system dynamics to construct a system dynamics evolutionary game rate variable-based in-tree model (referred to as the SD evolutionary game rate variable-based in-tree model) to analyze the feedback mechanism of stakeholders’ decision-making behavior in DT in different scenarios. The application of system dynamics more clearly showed the process of game players’ strategy evolution [45, 46].

### Simulation model of SD evolutionary game rate variable-based in-tree for DT

According to Theorems 1 and 2 of the SD evolutionary game rate variable-based in-tree model (see S1 Appendix), we combined the replicator dynamic equations of the three players, which calculated the evolutionary game system flow levels and flow rates as follows:

$x,\frac{\partial x}{\partial t}$: The proportion and variation of PC choosing to ‘empower’ in the process of DT.

$y,\frac{\partial y}{\partial t}$: The proportion and variation of NE choosing to ‘transform’ in the process of DT.

$z,\frac{\partial z}{\partial t}$: The proportion and variation of GA choosing to ‘supervise’ in the process of DT.

Therefore, the set of flow levels and flow rates for the replicator dynamic equations of the three-player evolutionary game is $\left[\left(x,\frac{\partial x}{\partial t}),(y,\frac{\partial y}{\partial t}),(z,\frac{\partial z}{\partial t}\right)\right]$, and the remaining parameters are exogenous variables.

**Fig 6:**
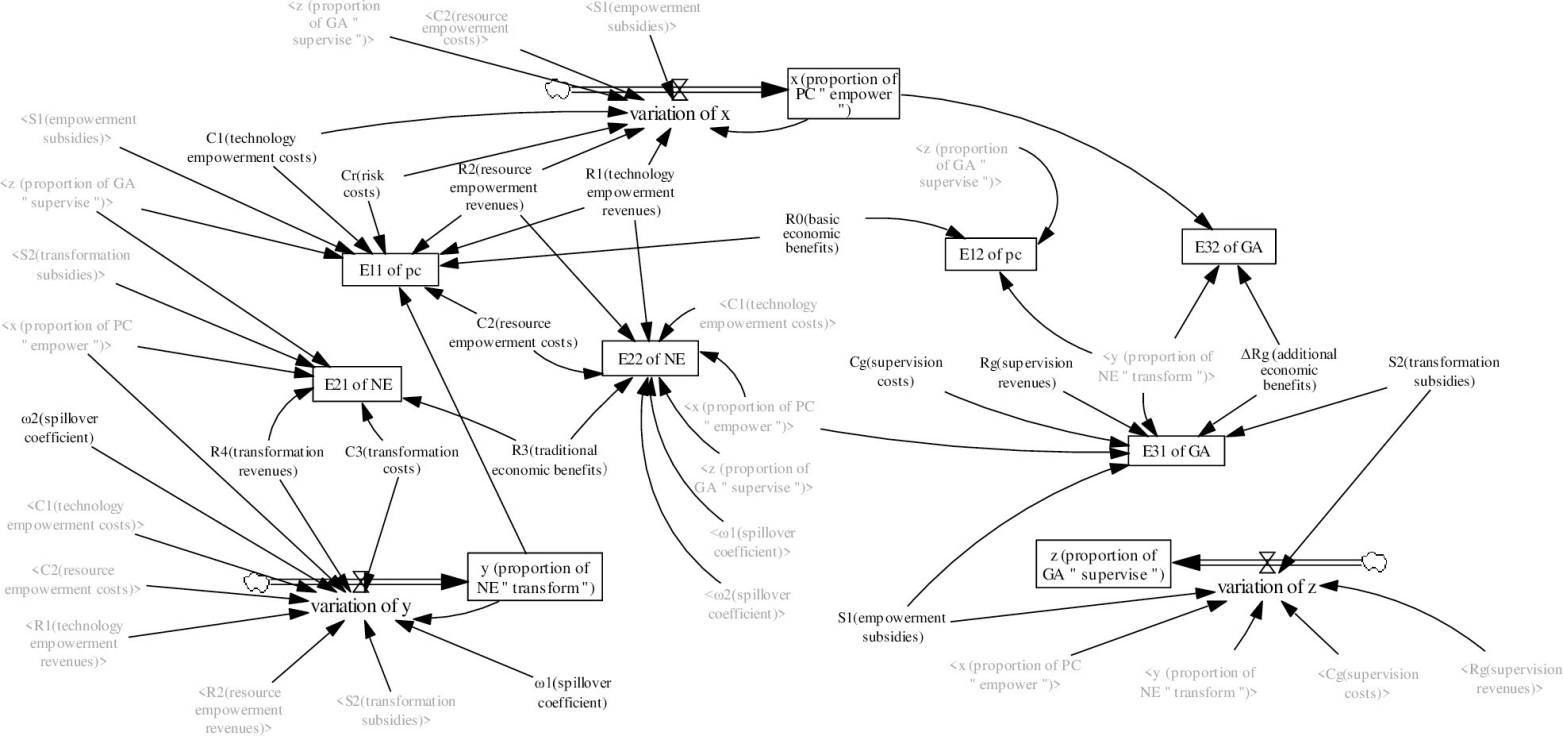
SD evolutionary game rate variable-based in-tree model for digital transformation.

We subsequently used Vensim software to construct the model, as shown in Fig 6. It is clear from the figure that the impact factors of the proportion of PC, NE, and GA in the digital innovation ecosystem.

## Results and discussion

After analyzing the stability of the system strategy, we set initial values for the proportion of players’ decisions as well as for the exogenous variables in the model. In this paper, we referred to the ways of setting the parameters of digital transformation, government regulation, and spillover effect in the relevant literature [41, 47, 48]. We set the initial values of the parameters according to the relevant digital transformation policies and the actual situations, as shown in Table 4.

**Table 4:** Initial key parameter values.

**Key parameter**	*R*_1_	*C*_1_	*ω*_1_	*ω*_2_	*R*_2_	*C*_2_	*C*_r_	*R*_4_	*C*_3_	*R*_*g*_	*C*_*g*_	*S*_1_	*S*_2_
**Initial value**	15	10	0.4	0.4	12	8	3	10	6	10	5	2	2

### Initial scenarios of the three players

In this paper, the parameter values in Table 3 were substituted into this model to obtain the evolutionary simulation results of digital transformation. However, since Vensim software could only perform simulation experiments in two dimensions, we used MATLAB to simulate to better show the simulation results.

**Fig 7:**
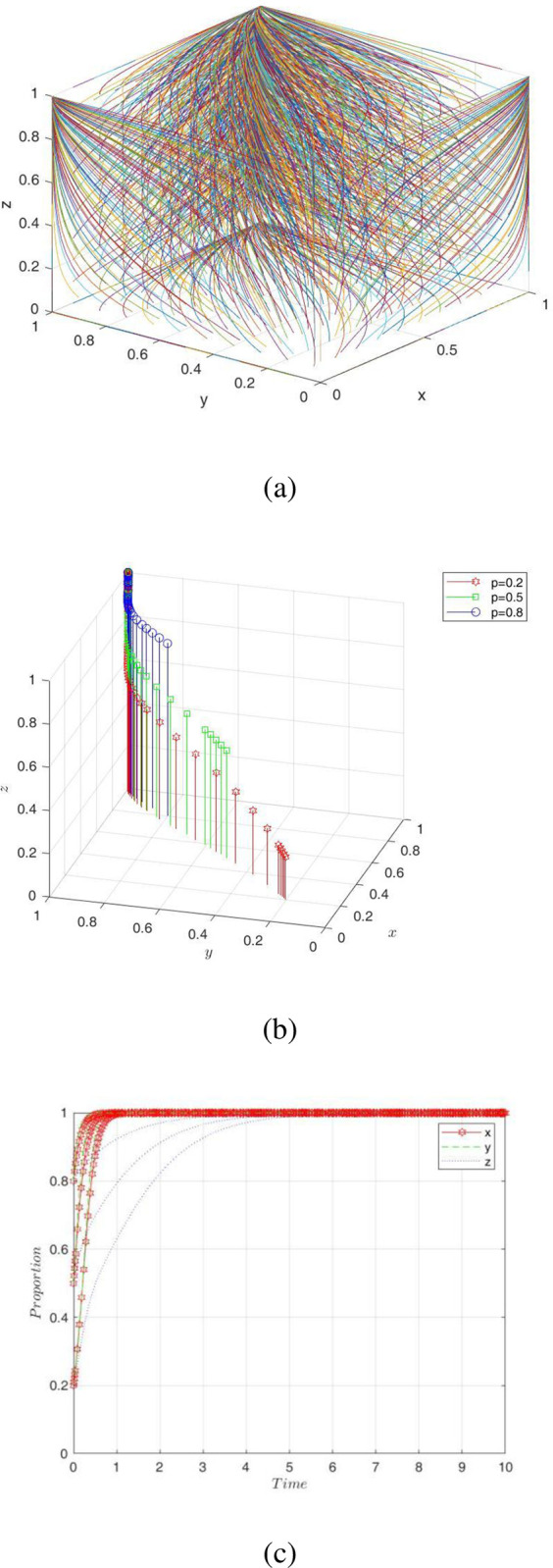
Simulation results of the initial evolutionary paths.

From Fig 7A, it can be seen that whatever values of x, y, and z were taken, they were eventually stabilized at ESS. To make the evolutionary path clearer, we show 3D and 2D graphs of the evolutionary paths of the PC, NE, and GA, respectively, in Fig 7B and 7C. The digital transformation system eventually stabilized at equilibrium point *E*_8_(1,1,1) when the initial willingness P was {*x* = *y* = *z* = 0.2;*x* = *y* = *z* = 0.5;*x* = *y* = *z* = 0.8}. In other words, a good business environment was created under the supervision of the government, which made it easier for PC to help NE transform. That is, they applied digital technology clusters to NE’s production and operation contexts and opened access to digital resources for them to achieve resource co-sharing and value co-creation. It was beneficial to improve the success rate of NE’s digital transformation and bring environmental and socioeconomic benefits to a country, forming a virtuous circle. Thus, the game strategies of the three players evolved over time and eventually stabilized at (empower, transform, supervise). It follows that digital transformation not only changes the operating model of enterprises but also leads to the development of cross-system relationships (e.g., government, research institutions, digital platforms, etc.), so the digital transformation strategy of enterprises is influenced by a combination of multiple actors [49]. Innovative companies such as Uber, Airbnb, Spotify, and Alibaba are successfully transforming themselves with advanced technologies and generating huge benefits, which has led to a trend of transformation among different types of companies that see the opportunity for digital transformation [50].

### Initial willingness change of the single player strategy

**Fig 8:**
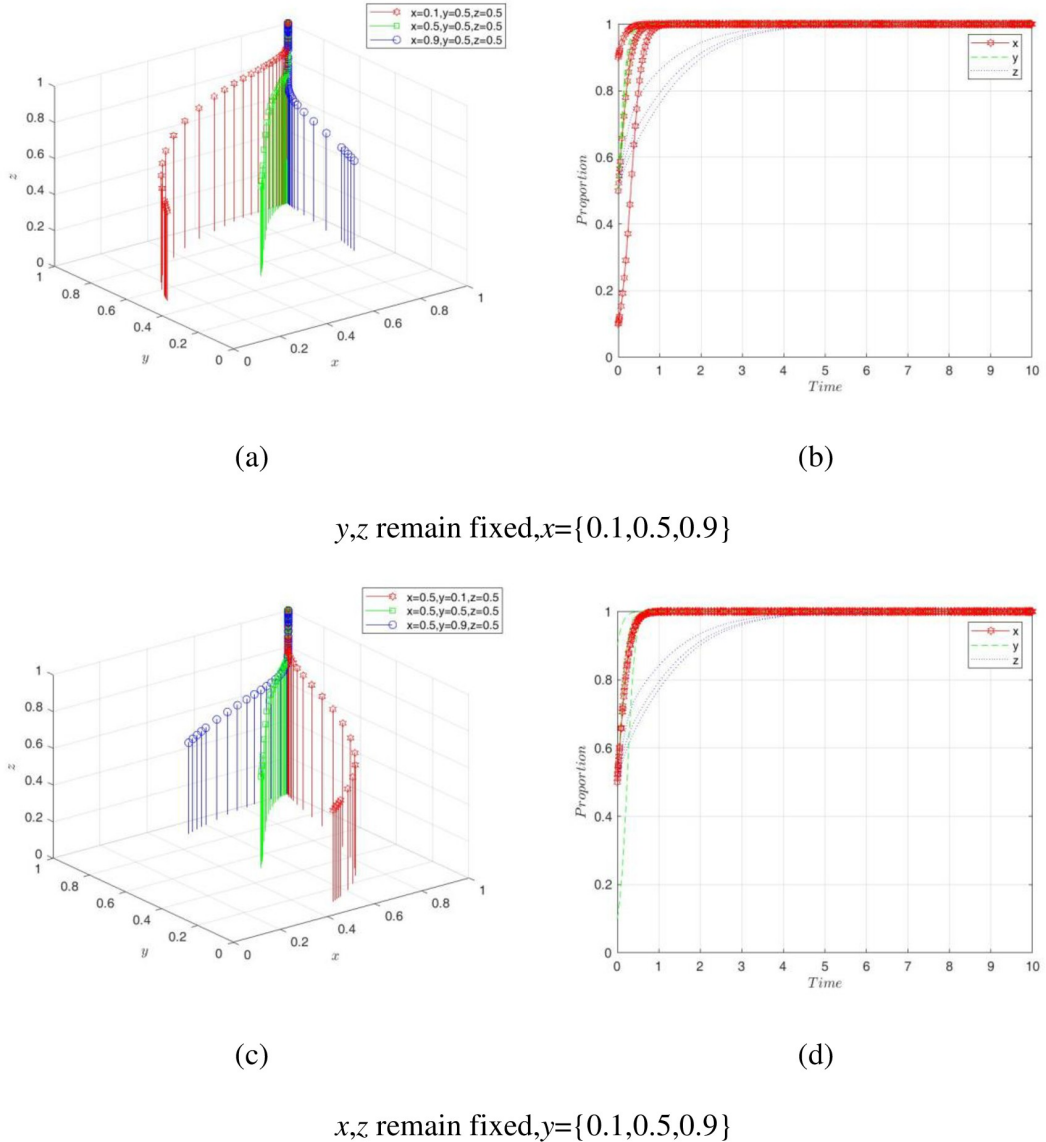
Simulation results of the initial willingness change.

In a game system, a player’s choice of strategy did not depend on any particular behavior but was jointly controlled by the players in the system. We simulated the initial willingness change of the three players and obtained the effect of the players’ game behavior on the evolutionary rate. Fig 8A and 8F show the simulation results of the evolutionary game under different initial willingness of the players. First, from the overall perspective, the system decision was ultimately stable at *E*_8_(1,1,1), regardless of the change in the initial willingness of the players. This result indicated that the three players, PC, NE, and GA, have high expectations for the economies of scale benefits generated by digital transformation. Thus, we have theoretically proven that digital transformation is a new trend in the development of the digital economy. Second, Fig 8A shows that when the initial willingness of NE and GA was kept fixed and the initial willingness of the PC was set to 0.1, 0.5, and 0.9, the combination of higher willingness {0.9, 0.5, 0.5} converged to ESS faster. Similarly, the same conclusion was obtained when the initial willingness of the NE and the GA changed. The results demonstrated that digital strategies not only benefit a single subject, but their positive externalities extend to all aspects of the economy and society so that an open and collaborative digital innovation ecosystem is shaped. The PC provides big data services or software development, and the GA promotes the construction of digital infrastructure, which makes the organic combination and application of new-generation information technology and traditional industries, giving rise to new models such as smart industries and smart cities [51]. Third, the change in the initial willingness of the PC had a greater impact on the government’s decisions compared to the nodal firms, as shown in Fig 8B and 8D. PC empowerment means that NE can reduce the resource consumption of underlying technologies, focus on cultivating professional capabilities, seize niche tracks, create business subplatforms, form a denser relationship network, and accelerate the process of improving the transformation support system of the government. Finally, Fig 8E and 8F indicate that the convergence speed of the GA’s decisions to the ESS varies considerably under the different initial willingness. This is because the environmental and socioeconomic benefits to the GA had a hysteresis. The GA was less willing to promote digital transformation than the companies that gained economic benefits and long-term high-quality development through digital interaction. It can be seen that the GA should pay more attention to the digital field and effectively play a leading role.

### Sensitivity analysis

Based on the stability analysis of the game system, the decision behavior of PC was mainly influenced by the revenues *R*_1_ and costs *C*_1_ of digital technology empowerment; the NE decision-making was mainly impacted by the revenues *R*_4_ and costs *C*_3_ of DT and the static/dynamic spillover effect coefficients *ω*_1_ and *ω*_2_; and the GA decision-making behaviors were mainly impacted by the revenues *R*_*g*_ and costs *C*_*g*_ obtained from regulation and the subsidies *S*_1_ and *S*_2_. Thus, we analyzed the impact on the players’ game decisions and the game system by adjusting the magnitude of the key exogenous variables.

(1) Adjusting the key variables of the PC

**Fig 9:**
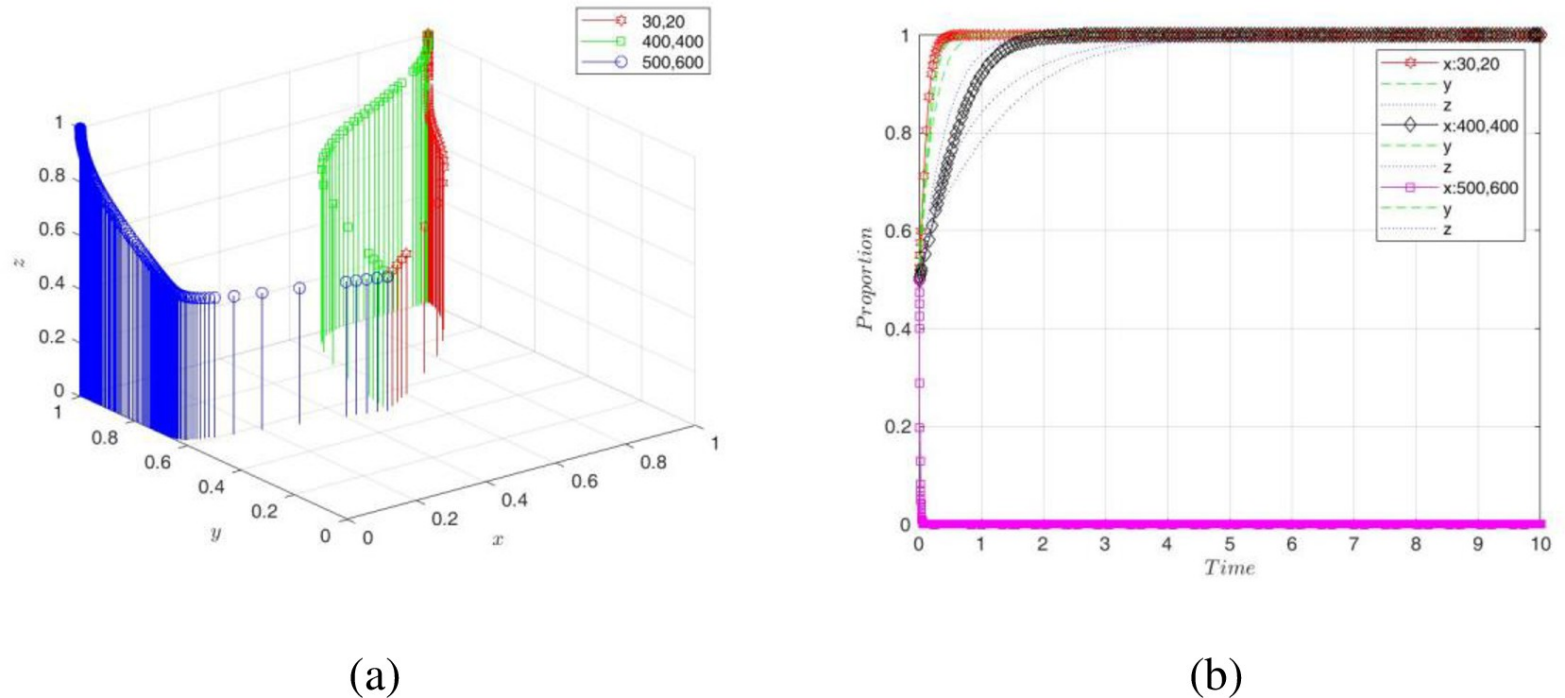
Impact of *R*_1_,*C*_1_ on PC decision-making and game system.

To observe the impact of digital technology empowerment revenues and costs of the PC on their game strategies and system stability, this paper assumed {R1, C1} = ①{30, 20}, ②{400, 400}, and ③{500, 600}, respectively, and the simulation results are shown in Fig 9. The study found that the lower the profitability of the platform centers through digital technology empowerment, the lower the willingness to choose the "empower" strategy and the slower the convergence to ESS. However, even if the profits of digital technology empowerment were zero, the PC would still eventually be digitally empowered. The reason was that the PC supported the digital transformation of enterprises through two paths: digital technology and digital resources, while the marginal costs of the latter were close to zero and the revenues were sustainable, and if the economic benefits generated were sufficient to compensate for the loss of digital technology empowerment, the game evolutionary strategy of the PC would converge to "empower". Moreover, the development of platform centers needs to be realized through interactions with ecological participants, and the balance and evolution of the complementary and dependent relationship between the two is the endogenous driving force of the digitalization process.

(2) Adjusting the key variables of the NE

**Fig 10:**
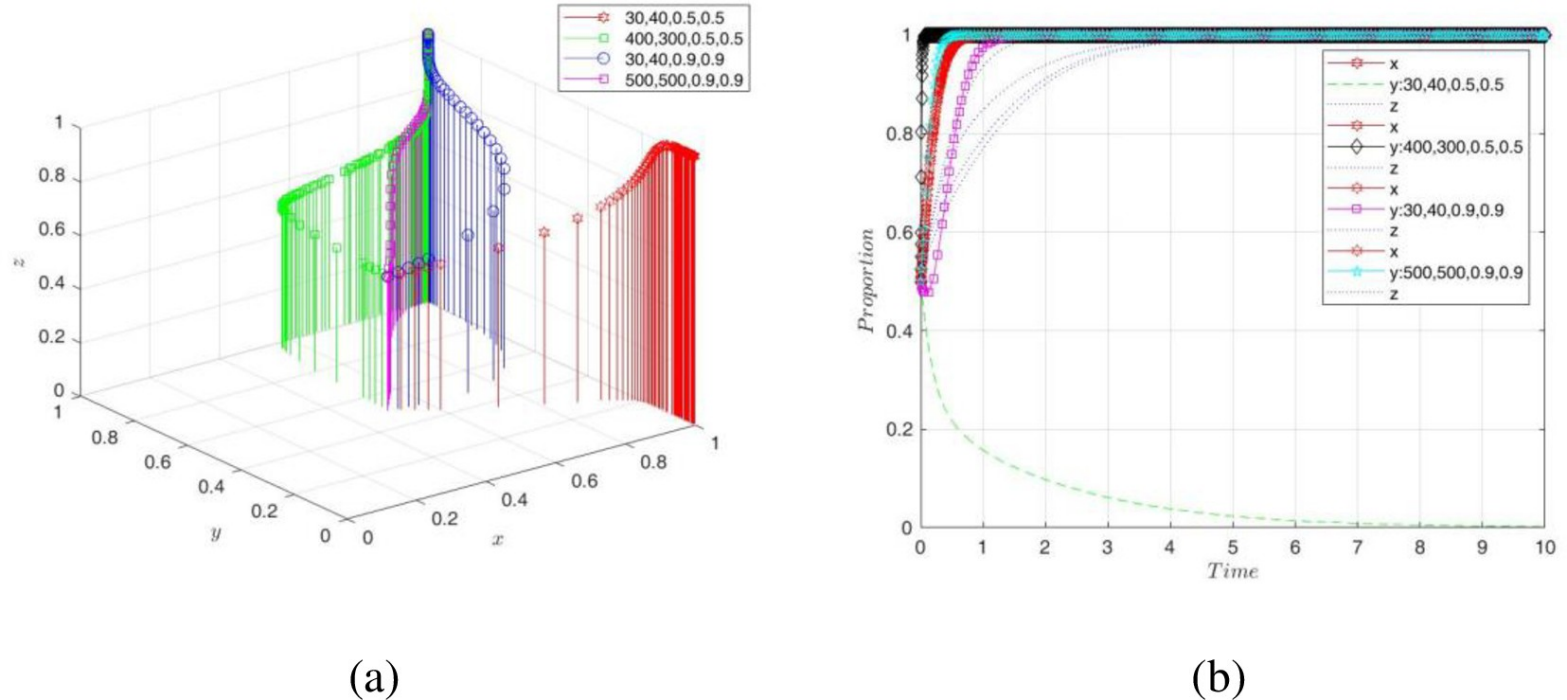
Impact of *R*4, *C*3, *ω*_1_, *ω*_2_ on NE decision-making and game system.

To observe the impact of digital transformation revenues *R*_4_ and costs *C*_3_ and static/dynamic spillover effect coefficients *ω*_1_, *ω*_2_ on the game strategies and system stability, this paper assumed{*R*_4_, *C*_3_, *ω*_1_, *ω*_2_} = ①{30,40,0.5,0.5},②{400,300,0.5,0.5}, ③{30,40,0.9,0.9}, ④{500,500,0.9,0.9}, respectively, and the simulation results are shown in Fig 10. It is found that when the digital transformation revenues of NE were not lower than the costs, the evolutionary game strategy was stable to "transformation" over time, namely, the higher the transformation revenues, the higher the motivation of NE to carry out digital transformation. Although the benefits of transformation were negative, when the positive externalities of digital technologies and the technological opportunities were large enough, NE still chose the "transform" game strategy. The reason was that data had become an important element of innovation in the context of the digital economy. The technical means of collecting, organizing, and storing data had made significant progress, accelerating the process of digital transformation of companies. Moreover, the spillover effect of digital technology and a large amount of data resources provide technology and resource support to NEs. In addition, digital transformation requires a large amount of capital investment and has a high financial risk. When decision-makers have higher digital sensitivity and a strong willingness to transform, they can better weigh the costs and benefits of the transformation process and allocate resources to collaborative R&D, equipment acquisition in renewal, and talent introduction, thus enhancing their risk-taking ability to achieve digital transformation [52].

(3) Adjusting the key variables of the GA

**Fig 11:**
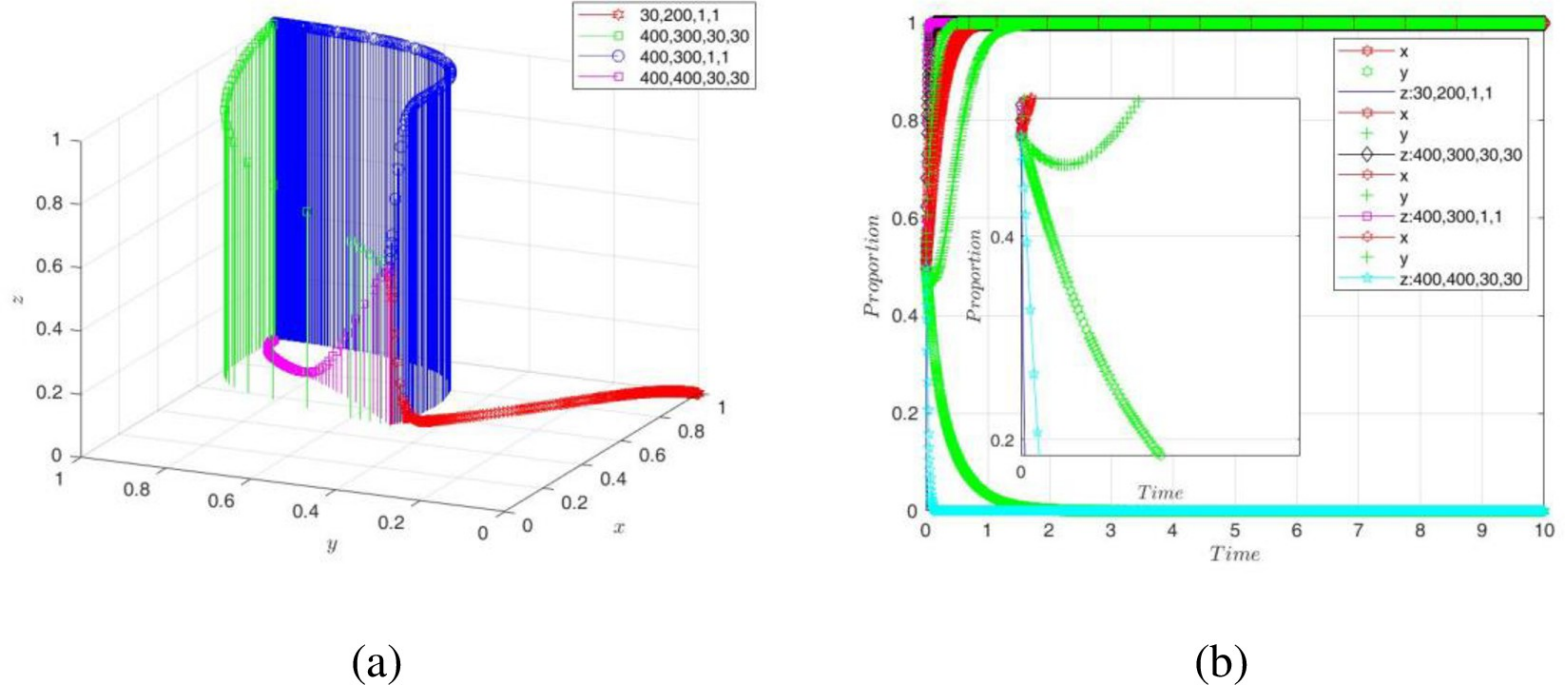
Impact of *R*g, *C*g, *S*_1_, *and S*_2_ on GA decision-making and game system.

To observe the effects of supervision revenues *R*_*g*_ and costs *C*_*g*_ and subsidies *S*_1_ and *S*_2_ on the game strategies and system stability, this paper assumed {*Rg*, *C*_*g*_, *S*_1_, *S*_2_} = ①{30,200,1,1}, ②{400,300,30,30}, ③{400,300,1,1}, ④{400,400,30,30}, respectively, and the simulation results are shown in Fig 11. It was found that when the government’s supervision revenues did not cover the costs and the subsidies, the government would have less incentive to supervise, and the evolutionary game strategy would stabilize at "nonsupervise". Additionally, when the digital transformation subsidy is 1, which is less than the initial value of 2, if the GA chooses "nonsupervise" at this time, it will reduce the enthusiasm of the NE for digital transformation, and their game strategy will eventually stabilize at "nontransform"; if the GA chooses "supervise" at this time, although the enthusiasm of the NE for transformation will decrease, they will still eventually choose the game strategy of "transform". The reason was that the GA continuously improved the digital governance system, promoted the establishment of digital infrastructure, and constructed a friendly digital environment, which promoted communication and cooperation among the parties in the system and created a good atmosphere for digital innovation. Although the node enterprises had extremely low subsidies for DT, they would still choose the game strategy of "transform" under a high-quality environment by government regulation. In contrast, if the GA did not supervise, they could not create a good digital atmosphere, and the meager subsidies could not support the DT of the NE. It fully explained that the digital transformation of traditional enterprises is led by the development strategy of the government, based on the radiation capacity of the new generation of digital infrastructure and platform technology, and formed the development goal of digital transformation and upgrading as a strategy.

## Comparative analysis of the model and simulation

Comparing the game model strategy stability analysis and simulation results, it is found that the platform center, node companies, and government agencies in the model stability analysis are always stable to the regular strategy. In the simulation, factors such as bounded rationality, information asymmetry, and exogenous variables are introduced, the trend of strategy evolution of the tripartite subjects is affected by the environment and the uncertainty of the subjects’ decisions, and the subjects’ willingness to make decisions shows random fluctuations. However, the overall trend of the simulation results is consistent with the results of the stability analysis of the replicated dynamic equations. The result also better reflects the credibility of the method in this study.

At the same time, the evolutionary trend of the game system proves that with the wide application of digital technologies (AI, blockchain, cloud computing, digital platforms), there is a strong willingness of enterprises to transform their business boundaries, production processes, structures, etc. [31] and reflects the lack of self-research and development motivation of NE [53], excessive digital investment [54], digital divide [55], imperfect regulations and policies [27] and digital platforms using technology to gain monopoly [56]. Therefore, the evolutionary game model and simulation study are valid.

## Conclusions and recommendations

In this paper, based on the bounded rationality of players, we apply evolutionary game and system dynamics methods to this study. Therefore, we establish a digital transformation SD evolutionary game model with PC, NE, and GA, and through the stability analysis of the game strategy and the simulation of the feedback mechanism, the following main conclusions are drawn:

1. From the overall results of the feedback mechanism simulation, government subsidies are the key factors influencing the players’ strategy evolution trajectories, and the revenues and costs generated by digital technology and digital resource empowerment, as well as the static/dynamic spillover effects of digital technology, affect the initial willingness of PC and NE, thereby influencing the stability of the system’s evolutionary strategy. From this, it can be seen that to solve the real problems of enterprises’ lack of willingness to digital transformation, insufficient motivation, and lagging progress in R&D, the government should give feedback to enterprises’ demands, introduce transformation guidance suggestions, increase investment in digital infrastructure construction and optimize local talent absorption policies to provide basic guarantees for improving the success rate of enterprises’ transformation.
2. From the initial willingness of the single subject strategy, regardless of how the initial willingness of the subjects changes, the system decision is finally stabilized at (enpower, transform, supervise), which indicates that the three players, PC, NE, and GA, have high expectations for the economies of scale benefits generated by digital transformation. We theoretically prove that digital transformation is the new development trend in the digital economy era. Compared to governments, the DT willingness of PC and NE is stronger. However, the change in GA willingness is the key to influencing the game system to converge to the equilibrium point. The willingness to transform is the first element in the constitution of the digital innovation ecosystem, but participants expecting a successful transformation need to weigh the investment of talent, technology, capital, and data and at the same time combine the characteristics of the industry and resource endowment and choose a comprehensive transformation approach and strategy.
3. From the impact of key exogenous variables on the evolutionary strategies, first, the lower the profitability of the PC through digital technology empowerment, the lower the willingness to choose the "empower" strategy, but the economic benefits of digital resources can cover the loss of digital technology, and the game evolution strategy of the PC will converge on "empower". Second, although the benefits of transformation were negative when the positive externalities of digital technologies and technological opportunities were large enough, NE still chose the "transform" game strategy. Third, when the government subsidies are less than the initial value of 2, the game system has two possible strategy choices: (empower, nontransform, nonsupervise) and (empower, transform, supervise). It is theoretically confirmed that the digital transformation of traditional enterprises presents the following characteristics, i.e., led by the government, based on innovation and dependent upgrading in cooperation with platforms, thus embarking on the track of digital transformation and upgrading.

Based on the above research findings, this paper makes the following recommendations:

1. Policy-makers should properly understand the role of governments in promoting DT, use intuitive methods to accurately assess the level of DT in a region, and quantify many different stages. Then, they can introduce relevant support policies and increase the number of subsidies for PC and NE, which will help accelerate the DT process. Platform centers need to pay attention to chips, cloud computing, artificial intelligence, and other basic technology R&D, but with increasing barriers to innovation, they need to increase investment in funds, talent, and equipment to make breakthroughs in core technologies. Meanwhile, they are supposed to enhance the efficiency of using data resources, protect the relevant rights in data transactions, and establish a sound management system for data privacy, security, and property rights to avoid the risk losses caused by the infringement of data information.
2. In the digital era, governments and other participants in the digital innovation ecosystem are no longer in a simple regulatory relationship but in a partnership based on data, information, and knowledge sharing. Therefore, governments can only bring a public value-creating ability to the digital economy to improve the digital transformation governance system, encourage real enterprises to recognize and implement digital innovation and accelerate digital transformation.
3. The PC should fully collect data resources in mobile terminals and communication networks so that data can flow reasonably and effectively. With the advantage that the marginal cost of data resources is almost zero, there is a possibility of an increasing scale effect, which effectively compensates for the loss of technology R&D and stimulates the willingness and vitality of collaborative innovation. As the barriers to technological innovation rise, the barriers to technology application fall. PC should increase R&D investment and enhance static/dynamic spillover effects, which can provide referenceable technical standards, technical opportunities, and an innovative atmosphere for the DT of NE.

## Limitations

First, this paper only constructs the three-player evolutionary game model of digital transformation, but as there are many actors in the digital innovation ecosystem, all of them will influence the ESS. Therefore, scholars can add scientific research institutions and other parties to construct a four-party evolutionary game model in future research. Second, we used the SD evolutionary game rate variable-based in-tree model to intuitively show the factors that influence the choice of digital transformation strategies, but the factors vary widely due to different contexts, such as political, economic, sociocultural, and technological development. Therefore, we suggest that future research increase the number of parameters to make the study more comprehensive. Meanwhile, this paper only used Vensim to draw the model and did not use it for numerical simulation, future research can combine Vensim and Matlab for numerical simulation and comparison. Third, scholars could use a variety of methods for comparative analysis to explore the differences in the conclusions, such as correlation analysis, fsQCA, and the differential game method. The first two methods can be used to explore the relationship between various influencing factors, and the differential game approach can be used to study the efficiency of the transformation in cooperative, follow, and non-cooperative situations. Finally, this paper does not include multiple DIEs as scenario cases for numerical simulation analysis, and future research can introduce more than two DIEs per region for case comparison analysis to improve the generalizability of the research results.

## Supporting information

S1 Appendix (DOCX)
