# Supplementary material for: An evolutionary game analysis of digital transformation of multiagents in digital innovation ecosystems
Source: PLoS One. 2023 Jul 21;18(7):e0289011. doi: 10.1371/journal.pone.0289011 (PMC10361489; doi:10.1371/journal.pone.0289011)
Supplement: S1 Appendix — (DOCX) [file pone.0289011.s001.docx]

**Supporting information**

**Appendix**

1.An arbitrary evolutionary game replicator dynamic equations, such as:

$$\left\{\begin{matrix} F\left(x_{1}\right)=\frac{\partial x_{1}\left(t\right)}{\partial t}=f_{1}\left[x_{1}\left(t\right),x_{2}\left(t\right),\cdots x_{n}\left(t\right),b_{1}\left(t\right),b_{2}\left(t\right),\cdots b_{m}\left(t\right)\right] \\ F\left(x_{2}\right)=\frac{\partial x_{2}\left(t\right)}{\partial t}=f_{2}\left[x_{1}\left(t\right),x_{2}\left(t\right),\cdots x_{n}\left(t\right),b_{1}\left(t\right),b_{2}\left(t\right),\cdots b_{m}\left(t\right)\right] \\ \vdots\\ F\left(x_{n}\right)=\frac{\partial x_{n}\left(t\right)}{\partial t}=f_{n}\left[x_{1}\left(t\right),x_{2}\left(t\right),\cdots x_{n}\left(t\right),b_{1}\left(t\right),b_{2}\left(t\right),\cdots b_{m}\left(t\right)\right] \end{matrix}\right.$$

**Theorem 1:** When *n*≥2, the replicator dynamic equations can be regarded as differential equations model under the flow level flow rate system, and the SD flow level flow rate system$\left\{\left(L_{1}\left(t\right),R_{1}\left(t\right)\right),\left(L_{2}\left(t\right),R_{2}\left(t\right)\right),\left(L_{3}\left(t\right),R_{3}\left(t\right)\right),\cdots,\left(L_{n}\left(t\right),R_{n}\left(t\right)\right)\right\}$ can be expressed as:

$\left\{\left(x_{1}\left(t\right),\frac{\partial x_{1}\left(t\right)}{\partial t}\right),\left(x_{2}\left(t\right),\frac{\partial x_{2}\left(t\right)}{\partial t}\right),\left(x_{3}\left(t\right),\frac{\partial x_{3}\left(t\right)}{\partial t}\right),\cdots,\left(x_{n}\left(t\right),\frac{\partial x_{n}\left(t\right)}{\partial t}\right)\right\}$.

**Theorem 2:** When *n*≥2, the replicator dynamic equations can be regarded as the SD rate variable based in-tree model. The model takes the flow rate variable as the root$\partial x_{i}\left(t\right)/\partial t$ and the flow level variable as the tail$x_{i}\left(t\right)$, and the flow level variable and exogenous variable can directly control the flow rate variable$\partial x_{i}\left(t\right)/\partial t$.
